# Rare Earth Elements in Testicular Function: Current Knowledge and Future Directions

**DOI:** 10.3390/ijms27167298

**Published:** 2026-08-15

**Authors:** Alessandra Santillo, Sara Falvo, Massimo Venditti, Maria Maddalena Di Fiore, Giulia Grillo, Gabriella Chieffi Baccari

**Affiliations:** 1 Department of Environmental, Biological and Pharmaceutical Sciences and Technologies, University of Campania “Luigi Vanvitelli”, 81100 Caserta, Italy; alessandra.santillo@unicampania.it (A.S.); mariamaddalena.difiore@unicampania.it (M.M.D.F.); giulia.grillo@unicampania.it (G.G.); gabriella.chieffi@unicampania.it (G.C.B.); 2 Department of Experimental Medicine, University of Campania “Luigi Vanvitelli”, 80138 Napoli, Italy; massimo.venditti@unicampania.it

**Keywords:** rare earth elements, testis, reproduction, lanthanides, cerium, gadolinium, lanthanum, neodymium, samarium, yttrium

## Abstract

Rare earth elements (REEs) are increasingly recognized as modulators of male reproductive health. This review presents the first comprehensive and critical analysis of the detrimental and potentially beneficial effects of REEs on male vertebrate reproductive function. In vivo and in vitro studies show that elements, particularly Gadolinium, Lanthanum, and Yttrium, share a toxicological profile characterized by oxidative stress, inflammation, mitochondrial dysfunction, endocrine disruption, and impairment of the blood–testis barrier. These mechanisms converge on both somatic and germ cell populations, ultimately compromising spermatogenesis and hormonal balance. Limited epidemiological data in humans are consistent with experimental findings, indicating inverse associations between seminal REE levels and sperm quality. CeO_2_ nanoparticles represent an exception, showing dose-dependent duality: although some murine studies describe toxic effects, a broader and more consistent body of evidence indicates their protective action by restoring testicular homeostasis in diabetic models, in rats exposed to pesticides, pharmacological agents or irradiation, and in in vitro-treated spermatozoa. Preliminary data on Yttrium (YO) and Gadolinium (GdVO_4_) nanoparticles similarly point to protective effects under pathological conditions, whereas Neodymium and Samarium show reproductive toxicity, including hormonal disruption and impaired sperm quality. Overall, REEs cannot be considered a homogeneous class: some pose reproductive hazards, others show potential biomedical utility. A systematic, mechanistic research framework is needed to resolve these dualities. It should contextualise reproductive risks in relation to the growing environmental and occupational exposure to REEs, while simultaneously exploring the biomedical potential of those elements that exhibit protective profiles.

## 1. Introduction

Reproductive health is a fundamental component of overall human well-being and a major global public health priority. Epidemiological data indicate that infertility affects approximately 17.5% of couples worldwide, with male-related factors accounting for nearly half of all cases [1].

The testis, which produces spermatozoa and secretes androgens, is central to male reproductive function. Proper spermatogenesis, from spermatogonia to spermatocytes and ultimately to mature spermatozoa, depends on metabolic and immunological support provided by testicular somatic cells and on testosterone synthesis by interstitial Leydig cells.

**Figure 1:**
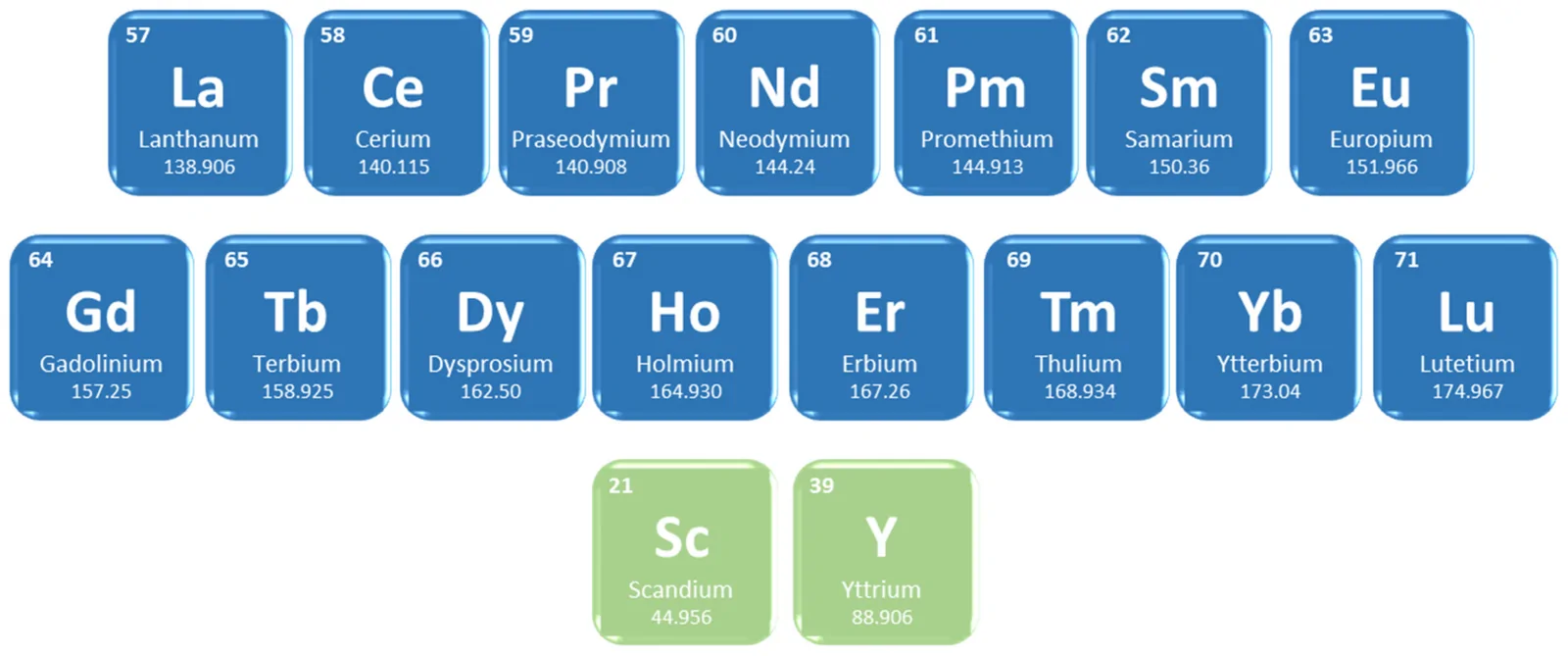
Rare earth elements with their atomic numbers, molecular masses, and symbols.

Male reproductive organs are particularly sensitive to environmental stressors, including toxicants and emerging pollutants [2,3,4,5,6,7,8]. Among these, rare earth elements (REEs) have attracted growing scientific attention in recent years, because of their rapid rise in global production and widespread use in advanced technologies. REEs comprise the fifteen trivalent lanthanides together with Scandium and Yttrium [9] (Figure 1).

The rapid expansion of advanced technologies is driving a substantial increase in the industrial production of REEs, which are widely used in catalysis, petroleum refining, hybrid vehicle batteries, magnetic materials, high-temperature superconductors, and nanoscale applications for catalysis, sensing, and biomedical imaging [10]. This growing demand has intensified mining, refining, and e-waste processing activities, leading to the environmental release of REEs into air, soil, surface waters, and sediments [11,12,13].

The intensification of REE-related industrial processes is also increasing occupational exposure, particularly through inhalation of REE-containing dusts, which has been associated with airway inflammation and fibrosis [14]. Additional exposure routes for the general population include ingestion, inhalation, and use of REE-containing products. Their prolonged persistence in biological systems and ability to bioaccumulate in human tissues further heighten health concerns [14]. In this context, environmental biomonitoring using organisms that selectively adsorb and accumulate REEs, such as lichens, mosses, plants, and microorganisms, has become an essential tool for detecting ultratrace concentrations, identifying emission sources, and assessing REE mobility and bioavailability in ecosystems [15]. This approach is crucial for understanding the environmental behavior of REEs as emerging contaminants and for supporting more sustainable environmental management strategies.

Accumulation of lanthanide complexes in the testis has been documented, and several studies have linked REE exposure to systemic and reproductive toxicity [16]. In aquatic organisms, lanthanides impair multiple physiological functions, including reproductive performance and fertilization success [17,18]. Galdiero et al. [19] reported that both Cerium and Erbium reduce the survival, growth, and reproduction of *Daphnia magna*. Gadolinium exposure leads to a considerable reduction in sperm motility and alters sperm morphokinetic parameters in *Mytilus galloprovincialis* [20].

Toxicological studies in vertebrates further demonstrate that Cerium, Gadolinium, Lanthanum, Neodymium, Samarium and Yttrium may induce oxidative stress, endocrine disruption, genotoxicity, and disturbances in spermatogenesis, raising concerns about their potential contribution to declining male fertility [14].

Human data further support the environmental relevance of these findings. Clinical studies have reported associations between seminal concentrations of Lanthanum, Cerium, and Gadolinium and altered sperm parameters, including reduced sperm concentration and changes in motility and morphology [21,22]. Moreover, Cerium ions (Ce^3+^) have been shown to induce oxidative damage and impair antioxidant defenses in spermatozoa. Conversely, CeO_2_ nanoparticles (CeO_2_ NPs) can be internalized by human sperm, causing significant DNA damage even at very low concentrations [23].

Taken together, the available evidence indicates that excessive exposure to REEs, together with their accumulation within the male reproductive system, may represent a relevant risk factor for male fertility. Nevertheless, scientific attention to the reproductive implications of REE exposure has grown markedly only in recent years, as evidenced by the sharp increase in publications over the past two decades.

This review provides the first comprehensive and critical evaluation of all experimental approaches that have explored the detrimental or, in some instances, potentially beneficial effects of REEs on male vertebrate reproduction. We systematically tabulate in vivo and in vitro studies, compare experimental designs, and critically assess how each model contributes to the current understanding of REE-induced alterations in reproductive processes. By integrating mechanistic insights with cross-species evidence, this review aims to clarify the state of knowledge, highlight inconsistencies, and define priority directions for future investigation.

## 2. Study Design and Method

A comprehensive literature review was conducted using PubMed (https://ncbi.nlm.nih.gov/pubmed (accessed on 8 August 2026)), the Scopus database (https://scopus.com), and Google Scholar (https://scholar.google.com), complemented by an examination of the reference lists of previously published articles and reviews. The review includes studies published from the earliest available reports up to 2025, with some additions from 2026. The literature from animal models was screened, with a specific focus on REE-associated effects on male reproductive health. References to clinical studies, when available, were included.

The search strategy used the following keywords: Rare Earth Elements, REEs, lanthanides, and individual lanthanide names, combined with male reproduction, male reproductive toxicity, testis, testicular toxicity, sperm, and spermatozoa.

A literature search performed in June 2026 across the PubMed and Scopus databases retrieved 2084 and 1303 articles, respectively. Total records screened: 3387. Google Scholar was additionally used to retrieve articles that were not accessible for download through the PubMed and Scopus databases. Duplicate records, review articles without original data, and studies unrelated to REE-associated effects on male vertebrate reproductive health were excluded.

All selected papers were evaluated according to the following criteria: use of animal model; availability of in vivo and/or in vitro data; clear description of the experimental procedures; specification of substances and their concentrations; exposure duration; measured parameters; and reported effects on the male reproductive system.

Records meeting the inclusion criteria: *n* = 75. Studies included in the review: *n* = 105.

## 3. Cerium

Cerium (Ce) is never found in nature in its pure metallic form, but it is the most abundant REE in the Earth’s crust. It is primarily extracted from two main minerals: monazite and bastnäsite. The element is mined and refined industrially to produce compounds such as CeO_2_.

CeO_2_NPs are among the most significant synthetic nanomaterials due to their wide-ranging agricultural and biomedical applications [24,25,26]. Their physicochemical properties and high environmental stability make them suitable for use in numerous sectors, contributing to an estimated global production of approximately 10,000 tons per year. Their use in automotive engines, high-temperature industrial processes, coatings, ceramics, and polishing agents generates emissions into the air and water, while their use in consumer products (cosmetics, sunscreens, polishes, and textile finishes) contributes to their dispersion through waste disposal, leachate, and incineration. The agricultural sector also contributes to contamination: fertilizers, pesticides, and biosolids derived from sewage sludge transport CeO_2_NPs into the soil, with potential transport to water bodies via surface runoff. Furthermore, the growing interest in the potential therapeutic applications of CeO_2_NPs, due to their antioxidant effect in cardiovascular, neurodegenerative, and oncological diseases [24,25], introduces additional release pathways, as wastewater treatment plants are unable to remove nanoscale residues effectively.

Several experimental studies indicate that CeO_2_NPs may pose a risk to male reproductive health [27]. Findings from in vitro germ, Sertoli, and Leydig cell models, as well as in vivo experimental studies, consistently show impaired testicular function. These outcomes, summarised in Table 1, support growing concerns regarding CeO_2_NP-induced reproductive toxicity.

In rats, repeated inhalation of CeO_2_ led to a marked accumulation of particles within testicular tissue, raising concerns about potential toxicological effects of chronic exposure to ambient air [28]. A pivotal contribution comes from the sperm genotoxicity study by Preaubert et al. [29,30], which demonstrated, for the first time, that extremely low concentrations of CeO_2_NPs can induce DNA damage in mouse and human spermatozoa, even in the absence of cellular internalization, suggesting a mechanism mediated by oxidative stress or surface interactions. These findings are consistent with the in vivo studies by Adebayo et al. [31] and Qin et al. [32], which demonstrated that mice exposed to CeO_2_ exhibited endocrine disruption, reduced levels of testosterone, FSH, LH, and prolactin, as well as an altered steroidogenic pathway, accompanied by pronounced oxidative stress, histological damage to seminiferous tubules, and compromised sperm quality. Testicular tissue alterations resulted in impaired sperm parameter quality, and reduced in vitro fertilization (IVF) rate and embryonic development were demonstrated in CeO_2_-treated mice [33].

The impact of CeO_2_NPs appears particularly relevant during early developmental stages. Specifically, Nemati et al. [34] showed that gestational exposure can disrupt the formation of the germ cell niche in newborn mice, reducing spermatogonia, Sertoli cells, and Leydig cells in a dose-dependent manner by increasing oxidative stress. These results are further supported by the organotypic model of Lee et al. [35], which demonstrated that CeO_2_NPs impair prepubertal spermatogenesis, inducing germ cell depletion and, at higher doses, damage to Sertoli and Leydig cells.

In contrast to the studies discussed above, in vivo toxicological models suggest beneficial and protective effects of CeO_2_NPs/Ce(NO_3_)_3_ on testicular function and sperm quality. For example, administration of a low dose of Ce(NO_3_)_3_ enhances succinate dehydrogenase (SDH), lactate dehydrogenase (LDH), sorbitol dehydrogenase (SODH), and glucose-6-phosphate dehydrogenase (G6PD) activities in the mouse testis [36]; in ageing rats, CeO_2_NPs stimulate hormonal function and spermatogenesis [37]. Furthermore, several studies converge on a clear antioxidant and anti-apoptotic role of CeO_2_. In particular, the study by Kobyliak et al. [38] revealed a significant increase in catalase (CAT) and superoxide dismutase (SOD) activities, associated with increased sperm count and improved quantitative sperm parameters after a 10-day administration of CeO_2_NPs in adult rats. The authors also reported an increased number and activation of Leydig cells, and a 2.5-fold rise in serum testosterone.

In vitro studies further corroborate the beneficial role of CeO_2_NPs in improving sperm quality. In Sarda ram spermatozoa, prolonged exposure to CeO_2_ enhanced semen preservation during storage, increasing motility and membrane integrity without elevating ROS levels or inducing DNA damage [39,40]. Similar improvements in viability and motility following CeO_2_ treatment were observed in Awassi ram [41] and Holstein bull spermatozoa [42], accompanied by increased activities of SOD, CAT, and peroxidase, along with reduced glutathione levels. In bucks (*Capra hircus*), CeO_2_ exposure further improved plasma membrane and acrosome integrity and was associated with a higher pregnancy rate [43]. Consistently, exposure to CeO_2_ in pregnant mice induced anti-apoptotic effects in neonatal testicular cells and upregulated genes involved in testicular maturation [44].

Furthermore, growing evidence indicates that CeO_2_NPs consistently protect male reproductive function across multiple pathological models, although the underlying mechanisms may vary by context. Specifically, co-administration of CeO_2_ with malathion restored sperm count, motility, and viability in rats, an organophosphorus pesticide commonly used in agricultural and non-agricultural settings and was associated with improved redox balance [45].

CeO_2_ exerts a protective function in irradiated mice by reducing damage to sperm and spermatogenesis [46]. Similar protective outcomes were observed against cyclophosphamide (CP)-induced testicular injury, where CeO_2_ reduced oxidative damage, apoptosis, and histopathological degeneration, likely through free radical scavenging and antiapoptotic properties [47]. CeO_2_ also mitigated the reproductive toxicity of fipronil (FIP), an insecticide, by decreasing lipid peroxidation, inflammation, and apoptotic signaling and enhancing endogenous antioxidant defenses [48]. Protective effects were further confirmed in diabetic rats, where CeO_2_ reduced malondialdehyde (MDA) contents, caspase-3, and BAX levels, as well as the BAX/Bcl-2 mRNA ratio, thereby attenuating testicular apoptosis and oxidative stress induced by diabetes [49]. Consistently, administration of CeO_2_NPs to diabetic rats was shown to attenuate the detrimental effects of diabetes on the sperm parameters, DNA integrity, and Nrf2 expression levels [50]. These studies provide a basis for determining the role of CeO_2_NPs in the context of diabetes.

Recently, Hassan et al. [51] demonstrated that CeO_2_NPs may act as a promising protective agent against boldenone (BOL), an anabolic–androgenic steroid that induces reproductive toxicity in male rats, by mitigating lipid peroxidation and inflammation and enhancing antioxidant defenses in testicular tissue.

In rats subjected to testicular torsion–detorsion (T/D), a severe urogenital condition caused by twisting of the spermatic cord, CeO_2_NP administration reduced ischemic testicular injury [52], oxidative stress, and improved spermatogenic indices in a dose-dependent manner, consistent with their CAT and SOD activities [53]. Moreover, CeO_2_NPs attenuated apoptosis by modulating key apoptotic markers [54].

Finally, a multigenerational study indicates that high-dose Ce(NO_3_)_3_ did not impair reproductive outcomes across two generations of rats [55]. Specifically, prolonged oral exposure to Ce(NO_3_)_3_ (30–270 mg/kg) produced no detrimental effects on reproductive performance in rats. Key fertility parameters, including sperm quality, mating and conception rates, implantation, and fetal outcomes, remained unchanged. Histopathological evaluation of reproductive tissues revealed no treatment-related lesions, supporting the conclusion that Ce(NO_3_)_3_ did not elicit reproductive or developmental toxicity under the tested conditions.

In conclusion, although a limited number of experimental studies have described adverse effects of CeO_2_NPs on testicular physiology, the overall body of evidence points to a more nuanced profile that includes clear beneficial actions. Short-term oral exposure to low doses of CeO_2_NPs (1 mg/kg for 10 days) appears to enhance testicular antioxidant defenses and support hormone production. In contrast, prolonged oral administration at higher doses (10–100 mg/kg for 35 days) or repeated intraperitoneal dosing (0.1–0.3 mg/kg over comparable periods) tends to elicit toxic outcomes, including reduced antioxidant capacity, inhibition of the steroidogenic pathway, decreased testosterone, LH, and FSH levels and impaired sperm parameter quality.

Overall, the dual protective-toxic profile of CeO_2_NPs seems to depend more on dose and exposure duration than on the route of administration. Extended treatments likely promote nanoparticle accumulation within the testis, leading to functional impairment.

Importantly, several experimental findings highlight the beneficial role of CeO_2_NPs in mitigating testicular damage induced by drugs, pesticides, or pathological conditions. Moreover, their use to improve sperm quality in livestock species has produced encouraging results, suggesting promising future applications in in vitro reproduction techniques.

## 4. Gadolinium

Among the rare earth elements, Gadolinium (Gd) has become one of the most environmentally relevant lanthanides due to its extensive use in modern medical imaging and industrial technologies. Its applications span magnetic resonance imaging [56], where Gd-based contrast agents (GBCAs) are routinely administered, as well as radar systems, compact discs, and microwave devices. The widespread clinical use of GBCAs, together with the growing deployment of Gd oxide nanoparticles (Gd2O_3_) in diagnostic and therapeutic contexts [57,58], has markedly increased anthropogenic Gd emissions into both aquatic and terrestrial ecosystems [12,59,60,61]. In nature, Gd occurs as a component of minerals such as gadolinite, monazite, and bastnäsite. Although Gd is naturally released through mineral weathering, concentrations detected in European surface waters now far exceed geochemical background levels, clearly reflecting the pervasive input of GBCA-derived Gd. Moreover, recent studies demonstrate that plants and aquatic organisms can readily take up Gd from contaminated waters [19,59,62,63], raising concerns about its potential transfer through the food web and the broader implications for environmental and human health.

Experimental evidence indicates that Gd, administered in various chemical formulations, acts as a potential disruptor of male reproductive function (Table 2).

An early study showed that repeated exposure to clinical doses of gadodiamide and gadoteric acid induced Leydig cell apoptosis, altered serum Ca^2+^ homeostasis, and reduced testosterone levels in adult rats, accompanied by impairments in spermatogenesis [64]. Consistently, biodistribution studies demonstrated that Gd can accumulate in testicular tissue following a single intravenous administration, persisting for weeks before slowly declining [65]. More recent work has expanded these findings to critical developmental windows: a single prepubertal exposure to gadoteric acid at clinically relevant doses caused long-lasting deficits in sperm production, motility, morphology, mitochondrial function, and chromatin integrity in adulthood, together with increased oxidative stress in testicular and epididymal tissues [66]. Complementary studies using oral GdCl_3_ and Gd_2_O_3_ in adult rats revealed a broad suppression of steroidogenesis, including downregulation of StAR, 3β-HSD, 17β-HSD, and 5α-reductase, alongside reduced testosterone and dihydrotestosterone (DHT) levels and decreased sperm number [67]. Mechanistic in vitro analyses in Leydig cells showed that Gd disrupts mitochondrial membrane potential (MMP), biogenesis, and dynamics, and impairs mitochondria-endoplasmic reticulum contact sites, triggering oxidative stress, autophagy, and apoptosis via inhibition of the Akt pathway [67].

A subsequent study demonstrated that Gd exposure also activated NF-κB-mediated inflammation as well as apoptotic and autophagic processes, and downregulated key spermatogenic markers (PCNA, pH3, SYCP3), ultimately leading to structural damage of seminiferous tubules and impaired sperm quality [68].

Finally, Sertoli cells represent a primary target of Gd toxicity [69]. In particular, TM4 cells exposed to GdCl_3_ or Gd_2_O_3_ exhibited reduced expression of the androgen receptor (AR) and junctional proteins, together with increased LDH activity. Moreover, Gd induced mitochondrial damage, impairment of Mitochondria-Associated Membranes (MAM), and triggered autophagic and apoptotic pathways [69]. Consistently, our recent experimental data indicate that exposure to GdCl_3_ or Gd_2_O_3_ induces significant alterations in the integrity of the blood–testis barrier (BTB) in rats (unpublished data).

An ultrastructural analysis revealed that Gd nitrate (GdNO_3_) was associated with phosphorus, appearing as electron-dense granules within the lysosomes of rat Sertoli and Leydig cells following i.p. administration [70]. A decrease in testosterone levels and an increase in sperm malformations contributed to reduced fertility in rats following GdNO_3_ exposure [71].

Interestingly, when Gd is incorporated into orthovanadate nanoparticles (GdVO_4_NPs), a chemically and biologically distinct formulation, its behavior differs markedly: in male rabbits under oxidative stress, GdVO_4_NPs improved sperm quality, normalized sex hormones, and enhanced antioxidant defenses [72].

Collectively, almost all experimental findings converge on a coherent model in which Gd, in various chemical formulations, acts as a multifaceted reproductive toxicant, capable of targeting both somatic and germ cells through oxidative stress, inflammation, mitochondrial dysfunction, and disruption of steroidogenic and proliferative pathways. An exception is GdVO_4_NPs, which instead exert protective or restorative actions under oxidative stress conditions. Nevertheless, while Gd emerges as a potential reproductive disruptor, the available data derive predominantly from high-dose animal studies, raising important questions about its relevance to environmentally realistic exposure levels.

## 5. Lanthanum

Lanthanum (La) and its nanomaterials are increasingly released into the environment due to their widespread use in industry, agriculture, and biomedicine, with over 900 kg/year emitted in Europe, far exceeding emissions of other toxic metals [73]. In nature, it occurs in cerite, monazite, and lanthanite, from which it is extracted by electrolysis of the chloride.

La compounds are used in catalysis, glass production, ignition systems, scintillators, electronics, and La carbonate is employed clinically as a phosphate binder. La_2_O_3_NPs are widely used in sensors, fuel cells, magnetic storage, and water treatment, thereby contributing to environmental dispersion.

La shows bioaccumulation in aquatic plants, mollusks, and fish, with potential trophic transfer [74]. Although ecotoxicity varies across studies, risks appear low in waters with pH > 7 and adequate alkalinity. Nonetheless, it can persist in ecosystems and reach humans via water, food, or inhalation. La_2_O_3_NPs can cross biological barriers, disrupt cellular homeostasis, and accumulate in multiple tissues, raising concerns about long-term exposure.

Emerging evidence indicates that La compounds exert measurable adverse effects on male reproductive function across experimental models and human observational studies (Table 2).

Early work already suggested direct interference by trivalent La ions with sperm physiology: in the presence of calcium, La^3+^ inhibited the initiation of rat sperm motility in a dose-dependent manner, indicating a disruption of calcium-dependent signaling essential for motility activation [75]. This foundational observation aligns with more recent in vivo findings showing that La-based nanomaterials can compromise testicular homeostasis. Studies using La_2_O_3_NPs have consistently demonstrated their ability to induce oxidative stress, inflammation, and structural damage within the testes [76]. Specifically, in mice exposed intragastrically for 60 days, La_2_O_3_NPs accumulated in the testes and increased lipid peroxidation and DNA oxidative damage, as evidenced by the MDA and 8-OHdG levels, and reduced SOD activity, indicating a pronounced redox imbalance. Concomitantly, alterations in the expression of BTB-related genes suggest that these nanoparticles can cross or destabilize the BTB, thereby amplifying their toxic potential [76]. A reduction in sperm count, motility, and survival was also induced by La_2_O_3_NPs. These findings collectively highlight a mechanistic framework in which oxidative stress and BTB impairment act as central drivers of La_2_O_3_NP-induced testicular dysfunction, with consequent impairment of sperm quality.

Longer-term exposure studies reinforce this interpretation. Ninety-day repeated intragastric administration of La_2_O_3_NPs resulted in impaired spermatogenesis, reduced testosterone and GnRH, and increased LH, indicating a disruption of the hypothalamic-pituitary-gonadal axis [77]. Ultrastructural analyses further revealed more severe testicular damage with NPs compared to larger microparticles (MPs), underscoring the enhanced reactivity and bioavailability of nanosized materials. Mechanistically, La_2_O_3_NPs inhibited the nuclear translocation of Nrf2 and downregulated its downstream antioxidant targets (NQO1, HO-1, GSH-Px), thereby suppressing a key cytoprotective pathway and promoting apoptosis [77]. Similar inhibition of the Nrf2/ARE axis was observed following chronic exposure to LaCl_3_, which also increased levels of ROS and peroxidation of lipids, proteins, and DNA, reduced antioxidant enzyme activities, and increased Keap1 expression [78]. Administration of a higher dose of LaCl_3_ altered testicular enzyme activities, including alkaline phosphatase (ALP) and nitric oxide synthase (NOS), reduced sperm count, and impaired sperm quality [79].

Lanthanum nitrate La(NO_3_)_3_ did not affect testicular function or induce genotoxicity [80,81].

Notably, human data provide an important environmental perspective. In a large cohort of fertile men, long-term exposure to PM_2.5_ and PM_10_ was associated with elevated concentrations of La and other REEs in semen [22]. These metals were inversely related to total sperm number and sperm concentration, suggesting that airborne particulate pollution may impair semen quality partly through alterations in seminal trace-metal composition.

Collectively, these findings support a coherent model in which La compounds, in both nanoparticulate and salt forms, compromise male reproductive health through oxidative stress, Nrf2 pathway suppression, BTB disruption, and endocrine imbalance. The convergence between animal studies and human epidemiological data suggests the potential reproductive risks associated with environmental La exposure.

## 6. Neodymium

In nature, Neodymium (Nd) is found primarily in the minerals monazite and bastnäsite as well as in some monazite sands. Nd compounds have gained increasing toxicological relevance due to their expanding industrial use and the consequent rise in occupational exposure. Nd-iron-boron (NdFeB) alloys, which form some of the strongest permanent magnets currently available [82], are extensively employed in miniaturized electronic devices and in high-power electromechanical systems. This widespread application has led to a parallel increase in the production and handling of Neodymium oxide (Nd_2_O_3_), typically obtained through molten-salt electrolysis of rare earth oxide mixtures [83]. In occupational settings, inhalation represents the primary route of exposure to Nd_2_O_3_ particulates, which can deposit in the pulmonary alveoli and subsequently translocate into the bloodstream. Once systemically distributed, Nd ions exhibit a propensity for tissue accumulation, raising concerns regarding their potential to induce chronic toxicity.

Recent studies provide increasing evidence that Nd_2_O_3_ can impair male reproductive function through both structural and molecular mechanisms (Table 2). In vivo experiments in C57BL/6J mice demonstrate a clear dose-dependent accumulation of Nd in the testes, accompanied by reductions in sperm survival and increased sperm abnormalities [84]. Histopathological alterations, including germinal epithelium disintegration, indicate substantial disruption of seminiferous tubule integrity. These structural changes are paralleled by declines in serum LH, FSH, and testosterone, together with reduced expression of key regulators of steroidogenesis and spermatogonial maintenance such as CYP11A1, PLZF, and STRA8, suggesting that Nd_2_O_3_ interferes with both hormonal homeostasis and early germ cell development [84].

Transcriptomic analyses further expand these findings. RNA-seq profiling of testicular tissue from Nd_2_O_3_-exposed mice reveals widespread dysregulation of mRNAs, miRNAs, lncRNAs, and circRNAs, with enrichment of differentially expressed genes in pathways directly related to spermatogenesis [85]. These findings point to a multilayered disturbance of gene regulatory networks, and several non-coding RNAs emerged as potential early biomarkers of Nd_2_O_3_-induced reproductive toxicity. Decreased sperm count and increased sperm deformities were observed in Nd_2_O_3_-exposed mice.

More recently, a mechanistic study, carried out both in vivo and in vitro using mice and the TM4 cell line, respectively, has identified a specific regulatory axis underlying BTB disruption [86]. Nd_2_O_3_ exposure upregulates the long noncoding RNA SNHG5, which binds to and reduces levels of insulin-like growth factor 2 mRNA-binding protein 1 (IGF2BP1), thereby destabilizing Occludin (OCLN) mRNA and protein as well as other proteins (ZO-1, N-cadherin, β-catenin) and compromising BTB integrity. This SNHG5/IGF2BP1/OCLN axis provides a plausible molecular explanation for the barrier dysfunction and increased permeability observed after Nd_2_O_3_ treatment and aligns with growing evidence that the BTB represents a relevant toxicological target for multiple REEs, including La, Y and Gd, thus offering a new research direction and mechanistic framework for REE-induced reproductive toxicity.

Together, early data indicate that Nd_2_O_3_ acts as a multilevel reproductive toxicant, affecting hormonal regulation, germ cell maintenance, meiotic progression, sperm morphology, and BTB stability. The integration of histopathology, endocrine disruption, and transcriptomic alterations highlights a complex toxicological profile and underscores the need for further research to clarify exposure risks and identify reliable biomarkers of early reproductive injury.

## 7. Samarium

Samarium (Sm), another member of the lanthanide series, is the fifth most abundant rare earth element and is present in REE-rich minerals such as monazite, bastnäsite, and samarskite. Despite an annual global production of about 700 tons and broad industrial use, including fertilizers, magnetic materials, and medical radioisotopes, its toxicological profile remains poorly defined. In humans, the total body burden is approximately 50 g, mainly stored in the liver and kidneys, with around 8 µg/L circulating in the blood. After entering the bloodstream, Sm distributes predominantly to the liver (≈45%) and to bone surfaces (≈45%), where it can persist for up to a decade [87].

Sm may exert adverse effects on male reproductive function, although its toxicological profile remains far less characterized than that of other REEs. Experimental studies in mice indicate that the testis is the target organ following oral exposure to Sm nitrate (Sm(NO_3_)_3_) (Table 2). Sm(NO_3_)_3_ administered accumulates in the testis [88] and leads to marked inhibition of LDH and alkaline phosphatase (ALP) enzyme activities [89], structural disruption of the seminiferous tubules, reductions in spermatogenic cells, and decreased sperm production [90,91,92]. Ultrastructural analyses further reveal mitochondrial swelling and vacuolization, compromised nuclear membrane integrity, and chromatin margination within germ cells, pointing to profound cellular injury [90,91,92]. These alterations are accompanied by increased germ cell apoptosis, together with upregulation of p53 and Bax and downregulation of Bcl-2, supporting the involvement of the p53-mediated mitochondrial apoptotic pathway in Sm-induced testicular damage [91].

Functionally, both acute and subchronic exposures impair sperm quality, with a reduction in motility and acrosome integrity and an increase in sperm malformations, particularly abnormalities of the sperm head, which appear to be the most sensitive endpoint [92,93]. These findings indicate that Sm disrupts not only the architecture of the testis but also the quality of spermatozoa, suggesting potential consequences for male fertility.

Human evidence, although still scarce, aligns with the experimental findings. In men diagnosed with oligo-astheno-teratozoospermia (OAT), seminal plasma concentrations of Sm correlated with altered sperm parameters [94]. Individuals of reproductive age with OAT exhibited higher Sm levels than controls, and Sm concentrations showed inverse associations with progressive motility and total sperm count.

Overall, the few experimental studies, all conducted by a single research group, support the view that oral exposure to samarium nitrate acts as a testicular toxicant, encouraging further investigations into its mechanisms and its relevance for human reproductive health.

**Table 2:** Effects of exposure to Gd, La, Nd, Sm in mammal models.

RERs	Test Model	Experimental Exposure	Main Effects	References
**Gadolinium**				
Gadodiamide or Gadoteric acid	Sprague-Dawley rat	intravenous administration 0.1 mL/kg b.w. for 5 weeks (4 times weekly)	increased apoptosis in the Leydig cells; increased serum Ca^2+^ levels; reduced T levels; loss of spermatozoa; immature germinal cell accumulation	[64]
Gadodiamide	Sprague-Dawley rat	intravenous administration 0.6 mmol/kg b.w. for 5 weeks (4 times weekly)	Gd testis accumulation after 1 h; Gd testis concentrations were reduced by 194-fold after twenty-weeks post administration	[65]
Gadoteric acid	Wistar rat (15 postnatal day)	intravenous administration 0.6 mmol/kg b.w. (single dose)	in 110 pnd rats: reduced testicular and epididymal weights; decreased daily sperm production; increased epididymal sperm transit time; altered sperm motility, viability, morphology, mitochondrial function, acrosomal and DNA integrity; increased oxidative stress markers and lipid peroxidation	[66]
GdCl_3_ or Gd_2_O_3_	Wistar rat	oral administration 10, 20, 40 mg/kg b.w. for 4 weeks	decreased steroidogenic-related protein (StAR, 3β-HSD, 17β-HSD, and 5α-Red) expressions, serum and testicular T and DHT levels, and spermatozoa concentration, viability and motility	[67]
	TM3 cells	5, 50, 500 μM GdCl_3_ 5, 50, 200 μM Gd_2_O_3_ for 24 h	cytotoxicity and reduced cell proliferation (decreased PCNA protein levels); decreased MMP, mitochondrial biogenesis (PGC1-α, NRF1, TFAM), fusion (MFN2), fission (DRP1) and MAMs (ATAD3, SOAT1, FACL4) marker expressions; increased oxidative stress, autophagy and apoptosis by inhibiting the Akt pathway	
GdCl_3_ or Gd_2_O_3_	Wistar rat	oral administration 10, 20, 40 mg/kg b.w. for 4 weeks	testicular inflammation (NF-κB activation and increased TNFα and IL-6 levels); increased oxidative stress (elevated TBARS levels), activation of apoptosis and autophagy; downregulation of spermatogenesis-related proteins (PCNA, p-H3, SYCP3, PRM2); altered seminiferous tubule morphology, compromised sperm quality	[68]
	GC1 cells	5, 200, 500 μM for 24 h	cytotoxicity and reduced cell proliferation (decreased p-ERK1/2 and PCNA protein levels); disrupted oxidative homeostasis and induced autophagy and apoptosis, likely through the inhibition of AKT signaling pathway	
GdCl_3_ or Gd_2_O_3_	TM4 cells	5, 50, 500 µM GdCl_3_ 5, 50, 200 µM Gd_2_O_3_ for 24 h	cytotoxicity and reduced cell proliferation (decreased PCNA protein levels); reduced AR and junctional (N-CAD, OCN, ZO-1) protein expressions; increased LDH activity; mitochondrial and MAM damage; triggered autophagic and apoptotic pathways	[69]
GdNO_3_	Wistar rat	intraperitoneal injection 112 mg/kg b.w. (total dose) for 2 weeks (every 2 days)	alteration of Sertoli and Leydig cells; their lysosomes concentrated Gd associated with phosphorus under insoluble electron-dense granules; decreased T levels and fertility rates; increased sperm malformations	[70,71]
GdVO_4_	tert-Butyl hydroperoxide-treated Hyla rabbit	oral administration 0.05–0.10 mg/kg b.w. for 14 days	improvement in sperm motility, reduction in abnormal sperm; increased T levels, decreased E_2_ levels; decreased oxidative load and increased antioxidant potential	[72]
**Lanthanum**				
La^3+^	Sprague-Dawley rat spermatozoa	0–10^−3^ La^3+^ in the presence of 1.27 or 2.54 mM Ca^2+^	dose-dependent inhibition of sperm motility initiation	[75]
La_2_O_3_NPs	Kunming mouse	intragastric administration 2.5, 5, 10 mg/kg b.w.	accumulate in testes; increased MDA and 8-OHdG levels; decreased SOD activity; impairing the BTB-related genes; decreased sperm count, motility, and survival; moderate inflammatory response and apoptosis	[76]
La_2_O_3_MPs		10 mg/kg b.w. for 60 days		
La_2_O_3_NPs	Kunming mouse	intragastric administration 5, 10, 50 mg/kg b.w.	decreased serum T and GnRH, and increased LH; severe testicular ultrastructural abnormalities; La_2_O_3_NPs treatment inhibited the translocation of Nrf-2 from the cytoplasm into the nucleus reducing NQO1, HO-1, GSH-Px expression	[77]
La_2_O_3_NPs		50 mg/kg b.w. for 90 days		
LaCl_3_	CD-1 (ICR) mouse	oral gavage 10, 20, 40 mg/kg b.w. for 90 days	up-regulation of lipids, proteins and DNA peroxidation; excessive ROS production levels; reduced activities of SOD, GSH-Px, GST, GSH; downregulated Nrf2, HO-1, GCLC, NAD(P)H, NQO1, PKC, PI3K expressions; upregulated Keap1 expression	[78]
LaCl_3_	mouse	intraperitoneal injection 25, 50, 100 mg/kg b.w. for 35 days (every 4 days)	reduced sperm count, damaged sperm quality; high doses of LaCl_3_ inhibited ALP activity, medium doses increased NOS activity	[79]
La(NO_3_)_3_	Sprague-Dawley rat	oral gavage 0, 10, 30, 90 mg/kg b.w.	no visible reproductive toxicity	[80]
La(NO_3_)_3_	NIH mouse	oral administration 562.5, 1125, 2250 mg/kg b.w. (two treatments with a 24-h interval)	no genotoxicity	[81]
	CHL cells	1.25, 5, 20 µg/mL for 24 h		
**Neodymium**				
Nd_2_O_3_	C57 BL/6J mouse	tracheal perfusion 62.5, 125.0, 250.0 mg/mL for 28 days	decreased sperm survival and number; increased head and tail sperm deformity; germinal epithelial disintegration; reduced serum LH, FSH and T levels; downregulated testicular CYP11A1, PLZF and STRA8 proteins; abnormal testicular mRNA/miRNA/circRNA/lncRNA expression levels	[84,85]
Nd_2_O_3_	C57BL/6J mouse	tracheal perfusion 62.5, 125.0, 250.0 mg/mL for 28 days	increased SNHG5 mRNA expression; reduced IGF2BP1 and OCLN mRNA/protein levels; reduced ZO-1, N-cadherin, β-catenin protein levels and consequent BTB impairement	[86]
	TM4 cells	62.5, 125.0, 250.0 mg/mL for 24 h		
**Samarium**				
Sm(NO_3_)_3_	mouse	oral administration 4–500 mg/L for 3 months	inhibition of testicular LDH and ALP enzyme activities	[89]
	mouse	0, 5, 50, 500, 2000 mg/L for 90 days	testis accumulation (50, 500, 2000 mg/L treatment); at 500, 2000 mg/L doses: morphological testicular damage; decreased sperm motility and count; lower sperm acrosomal integrity	[88,90,93]
	ICR mouse	intragastric gavage ≥ 2880.00-mg/kg (4 times at 2 h intervals) or drinking water ≥500.00 mg/L for 90 days	disorganized seminiferous tubules; decreased spermatogenic cells and sperm; mitochondrial swelling, fuzzy nuclear membranes, and marginated chromatin; increased spermatogenic cell apoptosis rate; up-regulation of p53 and Bax, and down-regulation of Bcl-2; decreased sperm motility and acrosome integrity; increased sperm malformation percentage	[91,92]

## 8. Yttrium

Yttrium (Y) is one of the most versatile heavy REEs, occurring naturally in minerals such as xenotime and monazite and typically co-extracted during REE processing. It is a rare transition metal with a predominant +3 oxidation state, forming stable oxides and halides and developing a protective oxide layer that limits further corrosion. In modern technology, Y plays a key role in enhancing the performance of electronic displays, high-temperature ceramics, superconducting materials, and advanced laser systems [95]. Its broad industrial use across electronics, aerospace, automotive engineering, medical imaging, and laser technology has led to increasing human exposure through food, environmental contamination, and specific medical applications. Moreover, environmental diffusion of Y has risen substantially due to the clinical use of Y-based contrast agents for magnetic resonance imaging [96]. Although often considered to have low acute toxicity, concerns are growing regarding long-term exposure, bioaccumulation, and the potential for adverse health effects, underscoring the need for a deeper understanding of its biological fate and toxicological impact. Despite this, the implications of Y exposure for human reproductive health remain largely overlooked.

Across available experimental models, Y displays a form-dependent toxicological profile, with markedly different biological behaviors between soluble Y salts and Y oxide nanoparticles (Table 3). Soluble Y^3+^ compounds such as YCl_3_ and Y(NO_3_)_3_ consistently demonstrate direct testicular cytotoxicity, with converging evidence from in vivo and in vitro models. YCl_3_ exposure induces pronounced testicular damage in both mouse and rat models, accompanied by activation of apoptosis and autophagy in Sertoli, Leydig, spermatogonial, and spermatocyte populations, while sparing necrotic and pyroptotic pathways [97,98]. Transcriptomic profiling identifies extensive dysregulation of genes involved in mitochondrial homeostasis and Ca^2+^ signaling, indicating that Y^3+^ toxicity is driven by a combination of mitochondrial dysfunction, ROS overproduction, and intracellular Ca^2+^ overload. Mechanistically, suppression of the Nrf2/HO-1 antioxidant pathway compromises redox defenses, while Ca^2+^ accumulation activates the CaMKII/AMPK axis, promoting programmed cell death. The independence of apoptotic and autophagic pathways suggests parallel, non-hierarchical activation of damage responses [97,98].

Long-term exposure to YCl_3_ decreases semen quality, gonadosomatic index, and testosterone levels [98]. The testicular toxicity appears to involve activation of the Ca^2+^/IP3R1/CaMKII pathway, and blocking IP3R1 or CaMKII mitigates Y-induced dysfunction in Leydig cells. This suggests a potential therapeutic avenue for Y-related testicular injury.

Chronic exposure to Y(NO_3_)_3_ increases ROS levels, induces DNA damage and activates the NF-κB pathway, with upregulation of pro-inflammatory cytokine expression, leading to impaired sperm motility, increased sperm apoptosis, and structural disruption of testicular tissue [99]. Both ROS scavenging (NAC) and NF-κB inhibition (JSH 23) effectively reverse these effects, confirming the centrality of oxidative stress-inflammation crosstalk in Y-induced reproductive injury. Expression of BTB-associated genes such as Claudin-11, OCN, and JAM was altered after high-dose Y(NO_3_)_3_ exposure, indicating the disruption of the crucial cell junctions needed for sperm production. Additionally, exposure to high-dose Y(NO_3_)_3_ reduced STAR and CYP11A mRNA levels and diminished SDH and LDH activities. An in vitro study confirmed the direct effects of Y(NO_3_)_3_ on both steroidogenesis and spermatogenesis [99].

Finally, a multigenerational study indicates that high-dose Y(NO_3_)_3_ (90 mg/kg) did not impair reproductive outcomes across two generations of rats [100].

Yttrium oxide nanoparticles (YONPs), which have low solubility and limited ionic release, exhibit protective activity against AgNP-induced reproductive toxicity, mitigating oxidative imbalance, apoptotic signaling, and sperm dysfunction in mice [101]. These findings suggest that inert oxide surfaces may buffer redox perturbations rather than directly interact with testicular cells.

Notably, Liu et al. [102] investigated the associations between exogenous metals in spermatozoa at single-cell resolution and human semen quality among 84 men. Single-cell mass cytometry demonstrated that Y accumulation in spermatozoa is highly heterogeneous and that this heterogeneity correlates negatively with sperm concentration and total sperm count. These findings suggest that Y may selectively accumulate in vulnerable sperm subpopulations, contributing to subtle impairments in semen quality not detectable through bulk metal measurements.

Overall, the experimental data suggest that Y salts interfere with male reproductive function through three principal mechanistic axes: oxidative stress with suppression of Nrf2-mediated defenses, intracellular Ca^2+^ dysregulation (IP3R1/CaMKII, CaMKII/AMPK), and NF-κB-induced inflammatory activation. In contrast, the only available experimental study reported protective effects of YONPs against AgNP-induced testicular damage.

**Table 3:** Effects of exposure to Yttrium in mammal models.

Yttrium	Test Model	Experimental Exposure	Main Effects	References
YCl_3_	C57BL/6 mouse	intraperitoneal injection 0, 25, 50, 100 mg/kg b.w./day for 21 days	increased apoptosis and autophagy; mitochondrial dysfunction; overproduction of ROS by impairing the Nrf2/HO-1 pathway; increased Ca^2+^ levels and activation of the CaMKII/AMPK signaling pathway	[97]
	TM3, TM4, GC1, GC2 cells	0, 100, 200, 400 µg/mL for 24 h		
YCl_3_	rat	oral administration 12, 24 mmol/L for 6 months	reduced semen quality, gonadosomatic index, and serum T levels; increased apoptosis; enhanced concentration of cytosolic Ca^2+^ and up-regulated expression of IP3R1/CaMKII axis	[98]
	Leydig cells	0, 3, 10, 30, 100, 300 mM for 24 h		
Y(NO_3_)_3_	BALB/c mouse	oral gavage 4, 20, 100 mg/kg b.w. for 30 days	reduced sperm motility, increased sperm apoptosis, disrupted testicular tissue structure and function; increased ROS, MDA, GSH, and activation of NF-κB pathway, upregulated pro-inflammatory cytokines; reduced mRNA levels of claudin-11, OCLN, JAM, StAR, CYP11A1, CYP17A1; reduced SDH and GSH activity; increased 8OH-DG protein expression	[99]
	TM3, GC1 cells	0, 50, 100, 200 μg/mL for 48 h		
YO-NPs	Ag-NPs-treated SWR/J mouse	intraperitoneal injection 40 mg/kg b.w. (weekly) for 35 days	YO-NPs minimized testicular damage induced by Ag-NPs (oxidative defense, sperm count and motility, apoptosis, and TBARS level)	[101]

## 9. Conclusions

**Figure 2:**
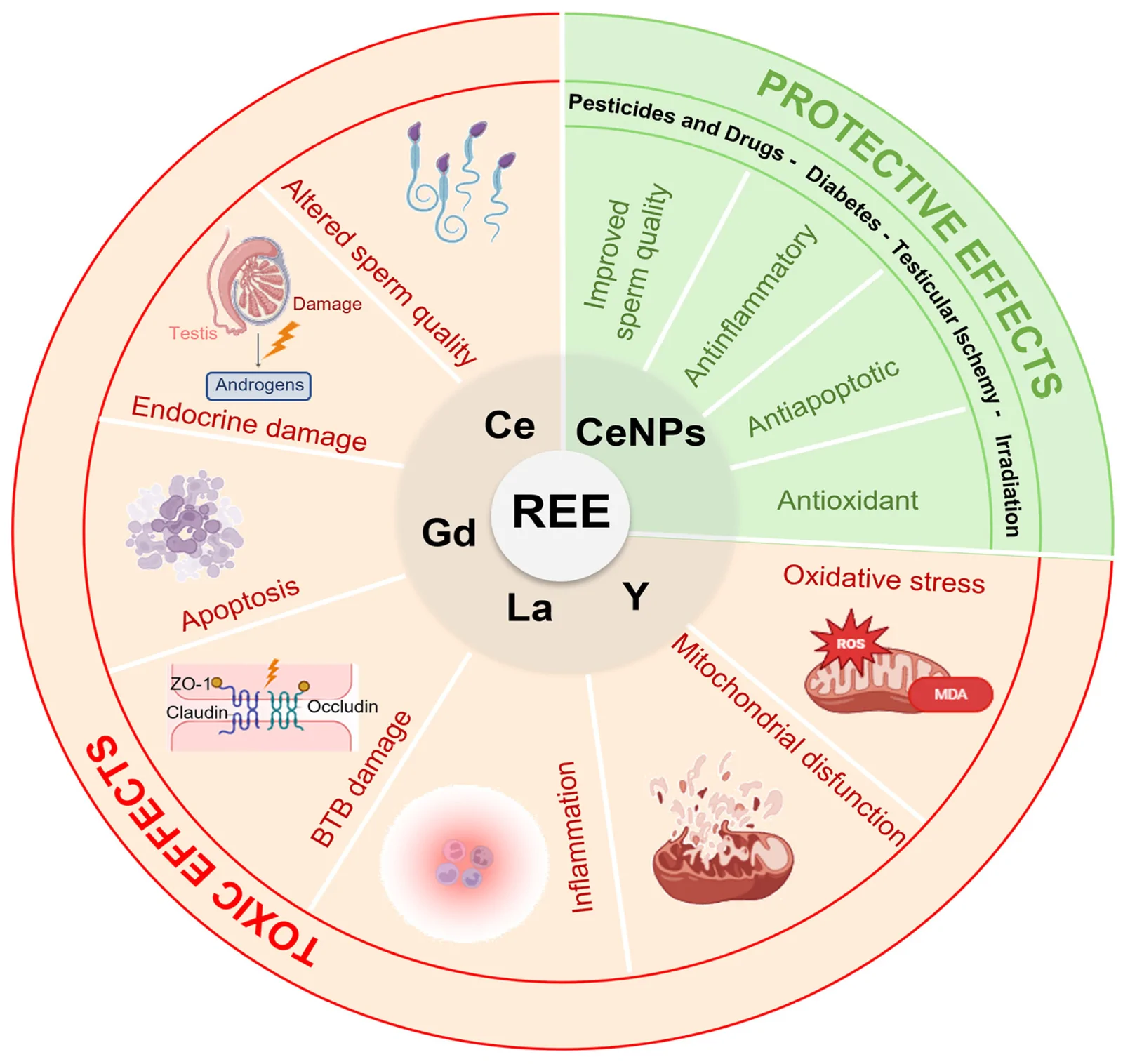
Toxic effects of Ce, Gd, La and Y on testicular physiology are mediated by oxidative stress, mitochondrial dysfunction, inflammatory responses, BTB impairment, apoptotic signalling, endocrine damage, and ultimately result in altered sperm quality. CeNPs exert a protective action by restoring testicular homeostasis in diabetic models, in rats exposed to pesticides, pharmacological agents or irradiation, and in in vitro-treated spermatozoa. Ce, cerium; Gd, gadolinium; La, lanthanum; Y, yttrium.

The toxicological assessment of REEs in reproductive physiology lags markedly behind the rapid industrial expansion and increasing market demand. Current knowledge is largely focused on Ce, Gd, La and Y, for which laboratory studies consistently indicate adverse effects on male reproductive health. These outcomes predominantly arise from alterations in testicular function induced by oxidative stress, apoptosis, inflammation, endocrine disruption, mitochondrial dysfunction, and BTB impairment, ultimately leading to compromised spermatogenesis and reduced sperm quality (Figure 2). Early epidemiological evidence aligns with these findings, correlating seminal concentrations of Ce, Gd and La with reduced sperm concentration and altered motility and morphology.

A notable exception is CeO_2_NPs, which display a dual and potentially protective profile (Figure 2). Short-term exposure to low doses appears to strengthen testicular antioxidant defenses and support hormone production, whereas excessive and prolonged exposure shifts this balance toward functional impairment. Moreover, several experimental models show that CeO_2_NPs can mitigate testicular injury induced by toxicants or pathological conditions. Additional promising evidence comes from studies in livestock, where CeO_2_NPs have improved sperm quality, suggesting potential applications in assisted reproduction. Preliminary data on Y (YO) and Gd (GdVO_4_) nanoparticles similarly point to protective effects in reducing the testicular damage induced by toxic agents or pathological conditions, though these observations require further validation.

Conversely, initial investigations on Nd and Sm reveal detrimental impacts on hormonal regulation and sperm quality, underscoring the need for deeper mechanistic exploration and careful evaluation of their reproductive risks. Significant knowledge gaps persist for several REEs, including praseodymium, dysprosium, europium, erbium and scandium, which lack mammalian data, even though reproductive toxicity in non-vertebrate organisms has been documented for some of them [16,103,104,105].

Overall, REEs do not constitute a homogeneous toxicological class: while several elements clearly pose reproductive hazards, others may offer opportunities for therapeutic or biotechnological innovation. A more systematic and mechanistically grounded research framework is needed to clarify these dualities, while parallel efforts should investigate the biomedical potential of REEs with protective profiles and integrate environmental monitoring to contextualise reproductive risks associated with industrial expansion and waste management.

## Figures and Tables

**Table 1:** Effects of exposure to CeO_2_ and Ce(NO_3_)_3_ in mammal models.

Cerium	Test Model	Experimental Exposure	Main Effects	References
CeO_2_	rat	inhalation for 28 days	testis accumulation after both 6 h and 28 days of exposure	[28]
CeO_2_	B6-CBA-F1 mouse spermatozoa	0.01 mg/L for 5 h	accumulation on plasma membrane; DNA damage; increased oxidative stress	[29]
CeO_2_	human spermatozoa	0.01 mg/L for 1 h	DNA damage	[30]
CeO_2_	BALB/c mouse	intraperitoneal injection 100, 200, 300 μg/kg b.w. for 5 weeks (thrice a week)	reduced T, FSH, LH, prolactin; increased testicular MDA; reduced antioxidant enzyme activities; reduced glutathione and nitric oxide levels	[31]
CeO_2_	C57BL/6J mouse	oral administration 10, 20, 40 mg/kg b.w. for 32 days	testis histopathological patterns and sperm DNA damage; decreased testis weight, sperm daily production and motility; reduced T levels and StAR, P450scc, P450c17, 3β-HSD, and 17β-HSD expressions; altered gene and protein SF-1 expressions	[32]
CeO_2_	NMRI mouse	oral administration 50, 100 mg/kg b.w. for 35 days	testicular tissue alterations; sperm DNA damage; decreased sperm parameter quality; diminishing IVF rate and in vitro embryonic development	[33]
CeO_2_	Pregnant NMRI mouse	intraperitoneal injection 10, 25, 80, 250 mg/kg b.w. on Gestational Day (GD) 7 and GD 14	in newborns: reduced number of spermatogonia, Sertoli, and Leydig cells; increased testis MDA levels	[34]
CeO_2_	testicular fragments from 5-day CD-1 mouse	10, 30, 50 μg/mL for 5 days	reduced number of both undifferentiated and differentiated germ cells; downregulated expression levels of their specific marker genes; decreased Sox9 mRNA/protein expression, and mRNA levels of AMH, Cyp17α1, HSD3b1 and Insl3	[35]
Ce(NO_3_)_3_	mouse	15, 30, 60 mg/kg b.w. for 60 days	low and medium doses: promoted LDH, SODH, SDH, G6PD testicular activities and cellular proliferation; inhibited apoptosis; higher dose shows adverse effects	[36]
CeO_2_	ageing rat	oral administration 1, 100 mg/kg b.w. for 10 days	stimulated hormonal function and spermatogenesis; higher dose had no significant effects	[37]
CeO_2_	rat	intragastric administration 1 mg/kg b.w. for 10 days	increased CAT and SOD activities; increased T levels, sperm count and quality; activation of spermatogenesis	[38]
CeO_2_	Sarda ram spermatozoa	22, 44, 220 µg/mL for 2 and 24 h	no effects on the redox status and on the DNA fragmentation level	[39]
CeO_2_	Sarda ram spermatozoa	44, 220 µg/mL for 96 h	enhanced sperm motility and velocity; beneficial effects on the integrity of plasma membranes	[40]
CeO_2_	Beetal buck spermatozoa	25, 50, 75, 100 µg/mL for 90 min	improved SOD, CAT, POD activities; reduced GSH; increased sperm total and progressive motility and velocity, plasma membrane and acrosome integrity and viability; enhanced pregnancy rate	[41]
CeO_2_	Awassi ram spermatozoa	25, 50 µg/mL for 72 h	improved sperm viability and motility; reduced sperm abnormalities	[42]
CeO_2_	Holstein bull spermatozoa	75, 125, 250 µg/mL	improved sperm viability percentage and motility	[43]
CeO_2_	Pregnant NMRI mouse	intraperitoneal injection 10 mg/kg b.w. on GD 7 and GD 14	in 6-day-newborns: decreased testicular mRNA expression of Bax, Caspase-3, and Gsk3-β genes, Bax/Bcl2 ratio; reduced apoptotic cells; increased Gata4, Sox8, and Rad54 mRNA/protein expression levels	[44]
CeO_2_	Wistar rat	intraperitoneal injection 15, 30 mg/kg b.w./day (co-treatment with malathion) for 28 days	restored malathion-induced testicular changes: protective effect on sperm counts, motility and viability; improvement of redox balance	[45]
CeO_2_	ionizing (2.5, 5, 10 Gy) irradiated-C57BL/6J mouse	intravenous administration 100 µM for 28 days (weekly)	preservation of the loss of spermatocytes and spermatids; reduced oxidative stress and DNA damage	[46]
CeO_2_	BALB/c mouse	intraperitoneal injection 100 μg/kg b.w./day (before the CP treatment) for 3 days	ameliorated testis CP-induced adverse effects: reduced caspase-3 immunoreactivity	[47]
CeO_2_	Albino rat	gastric gavage 35 mg/kg b.w. (co-treatment with FIP) for 28 days	diminished harmful impacts of FIP on testicular tissue: decreased lipid peroxidation, apoptosis and inflammation; increased antioxidant activities	[48]
CeO_2_	diabetic Wistar rat	intraperitoneal injection 30 mg/kg b.w./day for 2 weeks	attenuated testicular damage induced by diabetes: reduced apoptosis and oxidative stress, detrimental effects on sperm parameters, DNA integrity, and Nrf2 expression levels	[49,50]
CeO_2_	Albino rat (BOL-treated)	intraperitoneal injection 100 µg/kg b.w. for 8 weeks (twice a week)	diminished BOL detrimental effects on testicular tissue; reduced lipid peroxidation and inflammation; enhanced antioxidant activity	[51]
CeO_2_	Wistar rats subjected to testicular T/D	intraperitoneal injection 0.3 mmol/L (30 min before the cessation of ischemia)	diminished testicular ischemia–reperfusion injury	[52]
CeO_2_	Wistar rats subjected to testicular T/D	intraperitoneal injection 1, 2, 5, 10, 20, 50 mg/kg b.w. for 6 weeks (weekly)	reduced testicular T/D-damage: decreased testis oxidative stress; increased dose-dependent spermatogenic index	[53]
CeO_2_	Wistar rats subjected to testicular T/D	intraperitoneal injection 0.5 mg/kg b.w. (30 min before inguinoscrotal incision)	reduced testicular T/D-damage: decreased Bax, caspase-3, p53 proteins; decreased MDA levels; increased CAT and GST activities	[54]

## Data Availability

No new data were created or analyzed in this study. Data sharing is not applicable to this article.
